# Determinants of incidence trends in pancreatic neuroendocrine neoplasms

**DOI:** 10.1111/jne.70136

**Published:** 2026-02-03

**Authors:** Giuseppe Lamberti, Elisa Andrini, Adriana Di Odoardo, Arianna Zappi, Claudio Ricci, Davide Campana

**Affiliations:** ^1^ Department of Medical and Surgical Sciences (DIMEC) University of Bologna Bologna Italy

**Keywords:** healthcare access, incidence, neuroendocrine neoplasm, pancreas, SEER

## Abstract

The incidence of pancreatic neuroendocrine neoplasms (NENs) is rising; whether this reflects a true increase in disease occurrence or improved detection remains uncertain. We conducted a retrospective, population‐based study using data from the Surveillance, Epidemiology, and End Results (SEER) Program (1975–2021) to examine temporal trends in the incidence of pancreatic NENs and assess whether changes reflect improved detection versus a true increase. Incidence trends were stratified by demographic and socioeconomic proxies of healthcare access, including income, residential setting, and race as recorded in SEER at the county level. We identified 16,253 cases of pancreatic NENs (44.6% women; median age 62 years). Incidence increased 7.75‐fold between 1975 and 2021, rising from 0.21 cases per 100,000 population in 1975 to 1.58 per 100,000 in 2021. Median tumour size at diagnosis decreased significantly, with an average annual reduction of 0.73 mm (*R* ^2^ = 0.765; *p* < 0.001). After adjustment, incidence increased more steeply among men, individuals aged 40–65 years and >65 years (vs. <40 years), those recorded as White (vs. Black and other races), individuals with higher income, and those residing in urban (vs. rural) counties. Incidence also rose more steeply for tumours located in the pancreatic tail, for grade 1 tumours (vs. grades 2 and 3), and for smaller tumours (vs. larger ones). The rising incidence of pancreatic NENs is probably explained by improved detection, particularly among populations with greater access to healthcare, rather than by a true increase in disease occurrence.

## 1 INTRODUCTION

Neuroendocrine neoplasms (NENs) are a biologically and clinically heterogeneous group of tumours traditionally considered rare. Due to their low incidence and the challenges in diagnosis and classification, population‐based cancer registries, such as the National Cancer Institute's Surveillance, Epidemiology, and End Results (SEER) Program, are critical resources for studying their epidemiology.^1^, ^2^, ^3^ The SEER database, initiated in 1973 and currently covering approximately 46% of the US population, provides a robust framework for long‐term surveillance of cancer incidence and trends (https://seer.cancer.gov).

Latest data about epidemiology of NEN in the US leverages on SEER data from 1975 to 2021 and demonstrates a marked increase in both incidence and prevalence of NENs across all anatomical sites.^3^ Between 2000 and 2021, the incidence of NENs of the pancreas increased 4.3‐fold, placing the pancreatic NEN among NENs with the highest incidence (1.31 per 100,000 persons). Similarly, data from the US Cancer Statistics registry highlighted a sustained increase in the incidence of neuroendocrine tumours (NETs), particularly in pancreatic NETs, with an annual percentage change of 8.8, and early‐stage NETs, with an average annual percentage change in the study period of 5.2.^4^ Among gastrointestinal NETs, a sharper incidence rise is reported in males and older individuals.^5^

While the rising incidence of NENs is well documented, its underlying causes remain incompletely understood. A true increase in disease burden may be confounded by improved diagnostic imaging, increased use of cross‐sectional modalities, broader awareness, and evolving classification criteria. Yet, few studies have systematically disentangled these factors or examined incidence patterns in relation to tumour subtype or clinical characteristics. In particular, prior analyses often grouped heterogeneous NEN subtypes together, limiting insight into site‐specific trends.^4^, ^5^

To address this gap, we conducted a comprehensive, population‐based analysis by isolating pancreatic NENs using SEER data to clarify potential drivers of incidence changes. We aimed to characterise temporal trends in incidence compared with pancreatic ductal adenocarcinoma (PDAC) and to explore demographic and clinical correlates associated with these trends across key subgroups.

## 2 METHODS

### 2.1 Study design and data source

This was a retrospective, population‐based cohort study using data from the “1975‐2021 SEER Data (November 2023 Submission)” registry of the SEER Program, accessed through the SEER*Stat software. We included all patients diagnosed with pancreatic neuroendocrine neoplasms (NENs) and pancreatic ductal adenocarcinoma (PDAC) between 1975 and 2021. Only histologically confirmed cases were considered eligible.

Pancreatic NENs were categorised as well‐differentiated neuroendocrine tumours (NETs) or poorly differentiated neuroendocrine carcinomas (NECs) based on the histological classification already recorded in the SEER registry. No reclassification or modification of differentiation status was applied. Cases with non‐specific histology (i.e., “not otherwise specified” [NOS]) were excluded to ensure accurate pathological categorisation.

Tumours were identified using SEER ICD‐O‐3 topographic codes for the pancreas (primary site labelled: C25‐0/9). The following variables were extracted: age at diagnosis, sex, race (as reported in SEER), year of diagnosis, tumour location, histologic subtype, tumour grade, stage at diagnosis (localised, regional, distant), tumour size, initial treatment (surgery, chemotherapy, radiotherapy), survival status, residential setting (urban vs. rural), and area‐level estimated income. Cases with missing data were excluded from multivariable analyses.

### 2.2 Statistical analysis

Clinical and demographic characteristics of NEN and PDAC cases were compared using the chi‐square test for categorical variables and the Mann–Whitney *U* test for continuous variables, after assessment of normality by Kolmogorov–Smirnov test and visual inspection of histograms. Given the large sample size, only differences with effect sizes considered clinically meaningful were reported. Specifically, Cramér's V was used for nominal categorical variables, while the phi coefficient (φ) was applied in 2 × 2 contingency tables. For continuous variables, Cohen's *d* was used. Differences were considered clinically relevant when effect sizes were at least small, defined as Cramér's V or φ ≥ 0.10, and Cohen's *d* ≥ 0.20. Tumour size was analysed as both a continuous and a binary variable using the median value of all NEN tumours as cut‐off. County‐level household income data were obtained from the US Census Bureau via the National Historical Geographic Information System (NHGIS; https://www.nhgis.org). Median income values were extracted using the standardised B19001 table (household income in the past 12 months) from the 1980, 1990, 2000, 2010, and 2021 censuses and American County Survey (ACS) datasets, ensuring consistency in the definition of income bands across years. Each SEER registry county was linked to its corresponding income data using Federal Information Processing Standards (FIPS) codes (https://transition.fcc.gov/oet/info/maps/census/fips/fips.txt). Counties were then dichotomised into high‐income and low‐income groups using a threshold of $75,000 in inflation‐adjusted 2021 US dollars. This income variable was used to stratify incidence rates and evaluate socioeconomic disparities in the occurrence of pancreatic NETs over time. Area of residence at diagnosis was categorised as “urban” or “rural” using the US Department of Agriculture Rural–Urban Continuum Codes (RUCC) assigned to each county in the SEER registries. Because the RUCC classification system changed over time, we harmonised definitions across the study period. For diagnoses made from 1975 through 2002, we defined urban counties as those with RUCC codes 1 through 4, in accordance with the historical classification available in SEER for that period. For diagnoses from 2003 onward, urban areas were defined as counties with RUCC codes 1 through 3, reflecting the updated scheme. Counties with RUCC codes 4 through 9 were classified as rural throughout, and counties with RUCC code 3 (“metro areas of fewer than 250,000 population”) were consistently included in the urban category due to their proximity to urban healthcare infrastructure and higher likelihood of access to specialised care, consistent with prior epidemiologic studies.^6^, ^7^, ^8^, ^9^

Temporal trends in the incidence of NENs and the NEN‐to‐PDAC ratio were assessed using simple linear regression models. Additional stratified analyses were performed across subgroups defined by demographic and clinical variables (e.g., sex, age, tumour site, stage, grade, race as recorded in SEER, income, and geographic setting). Interaction terms (e.g., year × subgroup) were included to assess whether the rate of change over time varied by subgroup. To identify factors associated with a more pronounced increase in the incidence of NENs and the NEN‐to‐PDAC ratio over time, we performed pairwise linear regression analyses, comparing the slope of incidence trends between levels of each categorical variable (e.g., males vs. females, White vs. Black vs. Other, localised vs. metastatic stage). For each comparison, we estimated the annual change in the incidence of NENs and the NEN‐to‐PDAC ratio, along with standard errors and *p*‐values. Variables showing statistically significant differences in univariate analyses were entered into a multivariable linear regression model to assess independent associations with the change in incidence ratio over time. All analyses were performed using R statistical software (version 4.3.1). A two‐sided *p*‐value <0.05 was considered statistically significant.

## 3 RESULTS

### 3.1 Patient selection and characteristics

Data from 289,664 cases of pancreatic neoplasms were extracted. After excluding cases without pathology‐confirmed diagnosis (*n* = 42,273), those diagnosed as “unknown” (*n* = 15,955), and those with histology types other than those of interest (*n* = 16 adenocarcinoids, *n* = 27 mixed adeno/NEN tumours, *n* = 19,081 other pancreatic tumours), a total of 212,311 cases were included in the analysis. Of these, 196,058 cases were classified as PDAC (92.3%) and 16,253 as NENs (7.7%), including 8936 NETs (4.2%) and 7317 NECs (3.04%) (Figure S1). Compared to pancreatic ductal adenocarcinoma (PDAC), neuroendocrine neoplasms (NENs) were more frequently located in the pancreatic tail (40.7% vs. 16.3%) and less frequently in the head (40.1% vs. 67.8%), classified as grade 1 tumours (64.9% vs. 12.7%), and diagnosed at a localised stage (39.0% vs. 9.5%) (Table 1).

**TABLE 1:** Baseline characteristics of patients with pancreatic ductal adenocarcinoma (PDAC) and pancreatic neuroendocrine neoplasms (NENs), which include neuroendocrine tumours (NETs) and neuroendocrine carcinomas (NECs).

Variable	Category	PDAC (*n* = 196,058)	NEN (*n* = 16,253)	*p*	Effect size
Sex	Female	95,141 (48.5%)	7242 (44.6%)	<0.001	0.021 (φ)
	Male	100,917 (51.5%)	9011 (55.4%)		
Race	Black	21,040 (10.8%)	1712 (10.6%)	0.0013	0.008 (Cramer's *V*)
	White	158,599 (81.1%)	12,996 (80.4%)		
	Other	15,942 (8.2%)	1449 (9.0%)		
Site	Tail	24,308 (16.3%)	4995 (40.7%)	<0.001	0.178 (Cramer's *V*)
	Body	23,584 (15.9%)	2345 (19.1%)		
	Head	100,867 (67.8%)	4921 (40.1%)		
Grade	1	9972 (12.7%)	6663 (64.9%)	<0.001	0.428 (Cramer's *V*)
	2	34,229 (43.7%)	2147 (20.9%)		
	3	34,076 (43.5%)	1458 (14.2%)		
Stage	Diffuse	105,094 (56.1%)	6679 (42.5%)	<0.001	0.246 (Cramer's *V*)
	Localised	17,809 (9.5%)	6136 (39.0%)		
	Regional	64,493 (34.4%)	2899 (18.4%)		
Income	>$75,000	103,419 (57.5%)	9472 (60.1%)	<0.001	0.015 (φ)
	≤$75,000	76,510 (42.5%)	6279 (39.9%)		
Residential setting	Rural	21,377 (12.0%)	1642 (10.5%)	<0.001	0.013 (φ)
	Urban	157,487 (88.0%)	14,062 (89.5%)		
Age	Median (IQR)	69 (61–77)	62 (52–71)	<0.001	0.019 (Cohen's *d*)
Tumour size	Median (IQR)	38 (29–50)	30 (17–50)	<0.001	0.008 (Cohen's *d*)
Surgery	Yes	39,196 (20.3%)	7814 (48.4%)	<0.001	0.179 (Cramer's *V*)

*Note*: Data are *n* (%), unless otherwise indicated. φ = phi coefficient. Effect sizes are reported as Cramér's V for nominal variables, φ for 2 × 2 comparisons, and Cohen's *d* for continuous variables. Cramér's *V* or φ ≥0.10, and Cohen's *d* ≥0.20 are considered clinically relevant.

Abbreviation: IQR, interquartile range.

**FIGURE 1:**
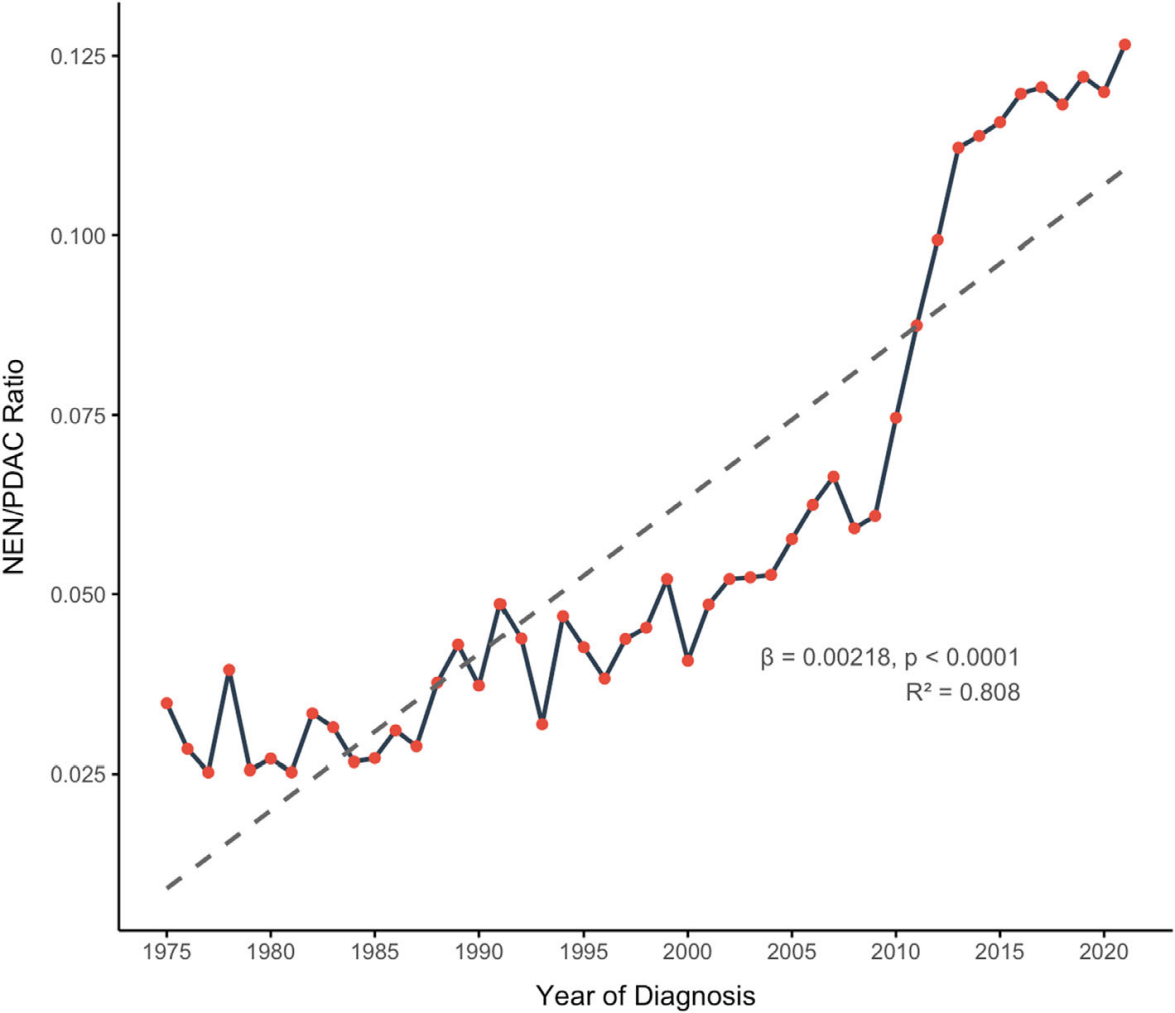
Temporal trend in the ratio of pancreatic neuroendocrine neoplasms (NEN) to pancreatic ductal adenocarcinomas (PDAC), NEN‐to‐PDAC ratio, from 1975 to 2021. The dashed line represents the fitted regression model (linear regression *β* = 0.00218, *p* < 0.0001; *R* ^2^ = 0.808).

We compared the annual number of NEN and PDAC cases using the NEN‐to‐PDAC ratio and observed a steady increase from 0.027 in 1980 to 0.127 in 2021, corresponding to an average annual increase of 0.00218 per year (*R* ^2^ = 0.808, *p* < 0.001) (Figure 1). The increase in the NEN‐to‐PDAC ratio over time was more pronounced for tumours located in the pancreatic tail compared to those in the head (*β* = 0.0055 vs. 0.0011, *p* < 0.0001), body (*β* = 0.0055 vs. 0.0026, *p* < 0.0001), and diffuse presentations. Similarly, the ratio increased more steeply among grade 1 tumours compared to grade 2 (*β* = 0.0441 vs. 0.0029, *p* < 0.0001) and grade 3 tumours (*β* = 0.0441 vs. 0.0011, *p* < 0.0001), and among cases diagnosed at a localised stage (*β* = 0.0111 vs. 0.0012, *p* < 0.0001) (Table S1).

### 3.2 NEN incidence

**FIGURE 2:**
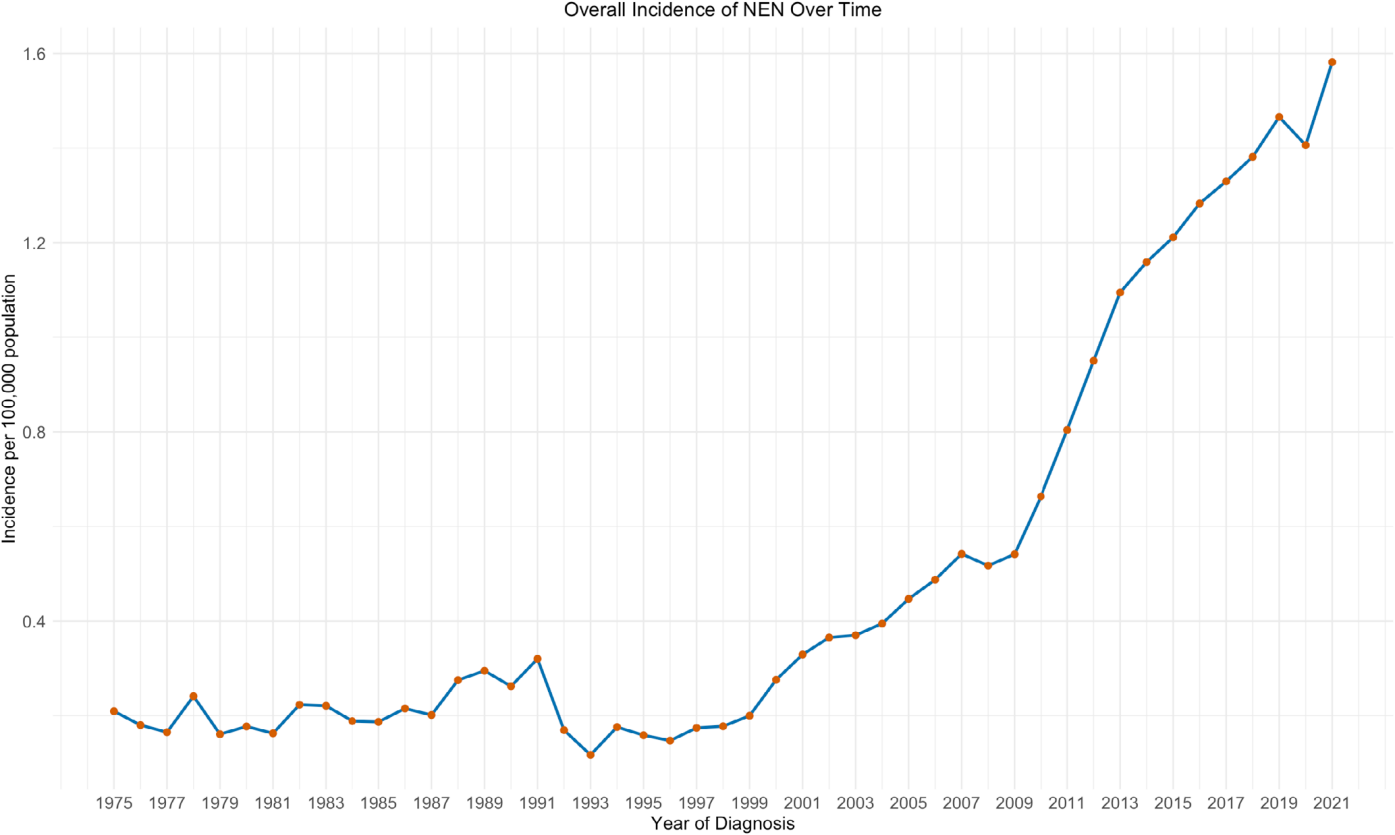
Annual incidence of pancreatic neuroendocrine neoplasms (NEN) from 1975 to 2021.

Overall, the incidence of NENs increased 7.75‐fold over the study period (1975–2021), rising from 0.21 cases per 100,000 population in 1975 to 1.58 per 100,000 in 2021, with a marked acceleration after 2010 (0.66 per 100,000 in 2010) (Figure 2).

When stratified by subtype, the incidence of NECs declined between 2010 and 2021 (from 0.52 to 0.35 per 100,000), whereas the incidence of NETs increased substantially over the same period (from 0.14 to 1.24 per 100,000) (Figure S2). This divergent trend possibly reflects the influence of the 2010 WHO classification and the subsequent introduction of the G3 NET category.^10^, ^11^

### 3.3 Analysis of trends over time

To evaluate whether the rise in NEN incidence could be attributed to improved diagnostic awareness and capabilities, we analysed temporal trends stratified by patient‐ and disease‐related characteristics.

The median tumour size at diagnosis significantly decreased from 50 mm in 1988 (the first year tumour size was recorded) to 25 mm in 2021, with an average annual reduction of 0.73 mm (*R* ^2^ = 0.765, *p* < 0.001), and a consistent downward trend since 2002 (Figure S3).

Within the overall increasing trend in incidence, we aimed to identify subgroups with steeper rises. To compare “small” and “large” NEN, tumour size was dichotomised by using the median value in the NEN population. In univariate analyses, incidence increased more markedly for localised tumours compared to those with regional or distant spread, for tumours located in the tail of the pancreas compared to other sites, for grade 1 tumours compared to grades 2 and 3, and for smaller tumours relative to larger ones (Table 2). Incidence also rose more in older age groups (>65 vs. 40–65 years, >65 vs. <40 years, and 40–65 vs. <40 years) and in people registered as White compared to those registered as other races.

**TABLE 2:** Univariate and multivariate pairwise comparisons of neuroendocrine neoplasms (NEN) incidence trends over time.

Covariate	Univariate			Multivariate		
	Estimate	SE	*p*	Estimate	SE	*p*
Sex male vs. female	s	0.0037	0.104	0.000031	0.000021	0.135
Age >65 vs. <40	0.0827	0.0077	<0.0001	0.000174	0.000029	<0.0001
Age >65 vs. 40–65	0.0469	0.0075	<0.0001	0.000002	0.000020	0.994
Age <40 vs. 40–65	−0.0358	0.0077	<0.0001	−0.00017	0.000028	<0.0001
Race White vs. Other	0.0094	0.0039	0.0179	0.000167	0.000046	<0.0001
Race White vs. Black	0.0047	0.0038	0.2212	0.000180	0.000034	<0.0001
Stage localised vs. distant	0.0059	0.0015	0.0001	0.000181	0.000027	<0.0001
Stage localised vs. regional	0.0098	0.0015	<0.0001	0.000183	0.000028	<0.0001
Site tail vs. body	0.0055	0.0011	<0.0001	0.000096	0.000031	0.0052
Site tail vs. head	0.0025	0.0011	0.0196	0.000089	0.000024	0.0005
Grade 1 vs. Grade 2	0.0123	0.0017	<0.0001	0.000155	0.000026	<0.0001
Grade 1 vs. Grade 3	0.0168	0.0015	<0.0001	0.000156	0.000028	<0.0001
Income >$75,000 vs. ≤$75,000	0.0137	0.0080	0.0935	0.000101	0.000023	<0.0001
Urban area urban vs. rural	−0.0041	0.0051	0.4190	0.000114	0.000033	0.0005
Size ≤30 vs. >30 mm	0.0079	0.0025	0.0023	0.000137	0.000023	<0.0001

**FIGURE 3:**
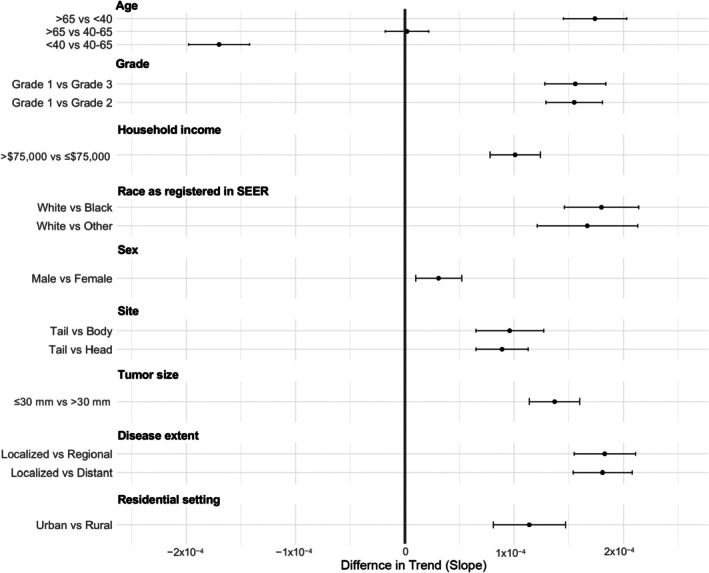
Forest plot of adjusted pairwise comparisons of incidence trends in pancreatic neuroendocrine neoplasms (NEN).

Multivariate analysis confirmed higher incidence increases among men, individuals aged >65 and 40–65 years compared to <40 years, individuals recorded as White compared to those recorded as Black and other races, those with higher income, and individuals residing in urban areas compared to those residing in rural areas. Additionally, the incidence rose more steeply for tumours located in the pancreatic tail, for grade 1 tumours compared to those of grade 2 and grade 3, and for smaller tumours compared to larger ones. Pairwise comparisons from the multivariate model are shown in Figure 3.

## 4 DISCUSSION

Our population‐based analysis confirms a sustained increase in the incidence of pancreatic NENs over the past decades.

This rise has been previously reported, yet the underlying causes remain incompletely understood.^3^, ^4^, ^5^ Hypotheses include improved diagnostic capabilities, increased awareness among clinicians, and a true rise in incidence potentially driven by environmental or lifestyle‐related factors. However, our findings suggest that the observed increase is predominantly attributable to improved detection rather than a true rise in disease burden.

In particular, the disproportionate increase in small, grade 1, localised NENs, especially those located in the pancreatic tail, and the greater rise in incidence among older patients, individuals recorded as White, those with higher income, and residents of urban areas point toward increased diagnostic activity as the primary driver of this trend.

The rising detection of small and localised NENs is consistent with the phenomenon of stage migration, which typically follows the adoption of effective screening programs or more sensitive diagnostic technologies. In the context of NENs, this is largely attributed to the increasing use of high‐resolution imaging modalities such as computed tomography (CT) and magnetic resonance imaging (MRI).^12^, ^13^ The pancreatic tail, often asymptomatic and inaccessible to first‐line imaging like ultrasonography, can be more easily explored thanks to these advanced modalities. This is reflected in the rising number of tail‐located tumours captured in recent years.

Although the use of functional imaging, particularly somatostatin receptor imaging (SSRI) with 68Ga‐DOTA‐peptides, has increased in recent years, its contribution to the rising incidence of pNENs is likely minimal. SSRI is mainly employed for staging or restaging and represents a third‐level investigation performed in referral centres rather than a routine diagnostic procedure; thus, it is improbable that its wider adoption has substantially influenced incidence estimates.

The disproportionately steeper increase in grade 1 NENs, compared with grade 2 and 3 tumours, that we observed in our analysis further reinforces the hypothesis of improved diagnostic sensitivity over time. It is now well established that the majority of small NENs, most of which are low‐grade, are indolent, with minimal risk of progression or clinical manifestation over a patient's lifetime.^14^, ^15^, ^16^ Prospective observational studies have consistently shown that fewer than 10% of pancreatic NET ≤20 mm in size exhibit measurable growth, and nodal involvement is exceedingly rare.^17^, ^18^ These observations are consistent with preliminary data from the prospective observational ASPEN study, which support the safety of active surveillance for pancreatic NETs ≤2 cm and indicate that, in the absence of worrisome features, upfront surgical intervention is not justified. The study further demonstrates that surgery remains feasible should such features develop and that metastatic progression, although possible, is exceedingly rare. These findings support the assumption that enhanced diagnostic capabilities have led to increased incidental detection of tumours that would have otherwise remained clinically silent.

Patient‐related factors also reinforce the diagnostic gain hypothesis. Older individuals are more frequently subjected to imaging procedures, often for unrelated reasons such as falls, cardiovascular events, or abdominal pain, potentially increasing the likelihood of incidental tumour detection.^12^, ^19^, ^20^, ^21^ Consistent with this, our analysis reveals a disproportionately steeper rise in the incidence of pancreatic NENs among individuals aged over 65 years.

Socioeconomic and geographic disparities further support this interpretation. Individuals with higher income and those residing in urban areas had a significantly greater increase in the incidence of pancreatic NEN. In the United States, access to advanced imaging is known to be influenced by socioeconomic status and urbanisation.^13^, ^22^, ^23^, ^24^, ^25^, ^26^ This discrepancy likely reflects differences in healthcare access and diagnostic resources rather than biological variation in disease occurrence.

Lastly, the higher incidence growth among individuals recorded as White compared to Black or other races may point to ongoing racial disparities in healthcare access, as previously documented.^26^, ^27^, ^28^

This study has several limitations. First, the SEER database, while comprehensive, is inherently limited by the accuracy and consistency of pathology reporting. Misclassification of tumour grade or histologic subtype is possible, especially given the evolving criteria across WHO classifications (2000, 2004, 2010, 2017, 2019). This is highlighted by the relatively high proportion of cases registered as NEC compared to those registered as NET and by the incidence trends of NET and NEC before 2010. To overcome this intrinsic limitation of the SEER registry, we considered all NEN in the analysis. Additionally, SEER data do not provide detailed information on clinical data, including performance status, functional status, biomarkers, or imaging modalities used for diagnosis.

Second, the long timeframe of the study, while allowing assessment of historical trends, introduces potential confounding due to changes in diagnostic criteria and imaging technologies over time. These shifts limit our ability to definitively exclude a true increase in incidence.

Nevertheless, the consistent and disproportionate rise in small, low‐grade, localised tumours, especially in subpopulations with greater access to advanced diagnostics, strongly suggests that the primary driver of increased incidence is improved detection.

## 5 CONCLUSIONS

In conclusion, the incidence of pancreatic NENs has increased steadily over the past four decades, with the most notable rise observed in grade 1 tumours, small lesions, those located in the pancreatic tail, and among individuals with likely greater access to healthcare, such as those with higher income, urban residency, and White race as recorded in SEER. While a true increase in tumour incidence cannot be entirely ruled out, our findings strongly support the hypothesis that the observed rise is largely due to enhanced diagnostic capabilities and increased clinical awareness of NENs.

## AUTHOR CONTRIBUTIONS

Giuseppe Lamberti and Davide Campana had full access to and verified the data. Giuseppe Lamberti and Davide Campana performed the statistical analyses and drafted the first version of the manuscript. Elisa Andrini, Adriana Di Odoardo, Arianna Zappi, and Claudio Ricci critically revised the manuscript for important intellectual content. All authors contributed to data interpretation, approved the final version of the manuscript, and agree to be accountable for all aspects of the work.

## FUNDING INFORMATION

This study received no funding.

## CONFLICT OF INTEREST STATEMENT

The authors declare no conflicts of interest.

## ETHICS STATEMENT

Data from SEER are de‐identified and do not require ethical approval for analysis or publication.

## PATIENT CONSENT STATEMENT

Data from SEER are de‐identified and do not require patient consent for use.

## Supporting information


**Data S1.** Supporting Information.

## Data Availability

The data underlying this study are available from the US National Cancer Institute's Surveillance, Epidemiology, and End Results (SEER) Program, November 2023 submission (1975–2021 dataset). Individual‐level SEER data are de‐identified and can be accessed by qualified researchers via SEER*Stat (https://seer.cancer.gov/seerstat/) upon completion of a data use agreement. County‐level socioeconomic and geographic variables used in this analysis were derived from publicly available sources: the US Census Bureau via the National Historical Geographic Information System (NHGIS; https://www.nhgis.org) and the US Department of Agriculture Rural–Urban Continuum Codes (https://www.ers.usda.gov/data-products/rural-urban-continuum-codes/). No additional patient‐level data beyond those publicly available will be shared. Statistical code used for the analyses is available from the corresponding author upon reasonable request.
