# Supplementary material for: Determinants of incidence trends in pancreatic neuroendocrine neoplasms
Source: J Neuroendocrinol. 2026 Feb 3;38(2):e70136. doi: 10.1111/jne.70136 (PMC12868397; doi:10.1111/jne.70136)

Supplementary Material

Determinants of incidence trends in pancreatic neuroendocrine neoplasms

**Table S1**	Temporal trends in the NEN-to-PDAC ratio across clinical, demographic, and tumor characteristics.	Page 2
**Figure S1**	CONSORT diagram of case selection.	Page 3
**Figure S2**	Annual Incidence Trends of pancreatic Neuroendocrine Tumors (NETs) and Neuroendocrine Carcinomas (NECs) from 1975 to 2021.	Page 4
**Figure S3**	Median primary tumor size by year of diagnosis of pancreatic neuroendocrine neoplasms (NEN).	Page 5

Table S1. Temporal trends in the NEN-to-PDAC ratio across clinical, demographic, and tumor characteristics.

Covariate	Comparison	Beta	p-value
Tumor Site	Tail vs Head	0.0055 vs 0.0011	<0.0001
	Tail vs Body	0.0055 vs 0.0026	<0.0001
	Tail vs Diffuse	0.0055 vs —	<0.0001
Grade	Grade 1 vs Grade 2	0.0441 vs 0.0029	<0.0001
	Grade 1 vs Grade 3	0.0441 vs 0.0011	<0.0001
Stage	Localized vs Distant	0.0111 vs 0.0012	<0.0001
Sex	Male vs Female	0.0023 vs 0.0020	0.165
Race	White vs Other	0.0024 vs 0.0022	0.495
Residential setting	Urban vs Rural	0.0033 vs 0.0026	0.107
Income	High vs Low	0.0035 vs 0.0027	0.041

NEN: neuroendocrine neoplasms; PDAC: pancreatic ductal adenocarcinoma.

Figure S1. CONSORT diagram of case selection.

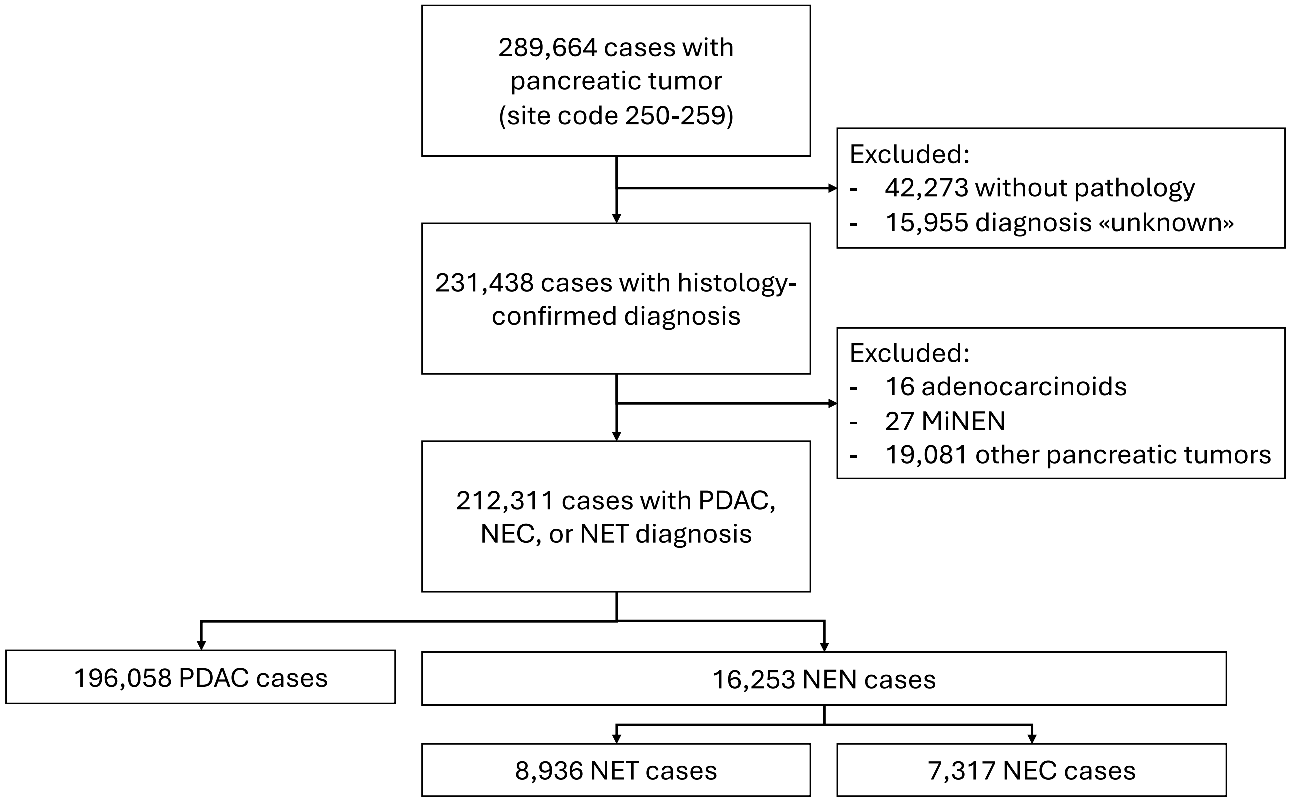

Figure S2. Annual Incidence Trends of pancreatic Neuroendocrine Tumors (NETs) and Neuroendocrine Carcinomas (NECs) from 1975 to 2021.

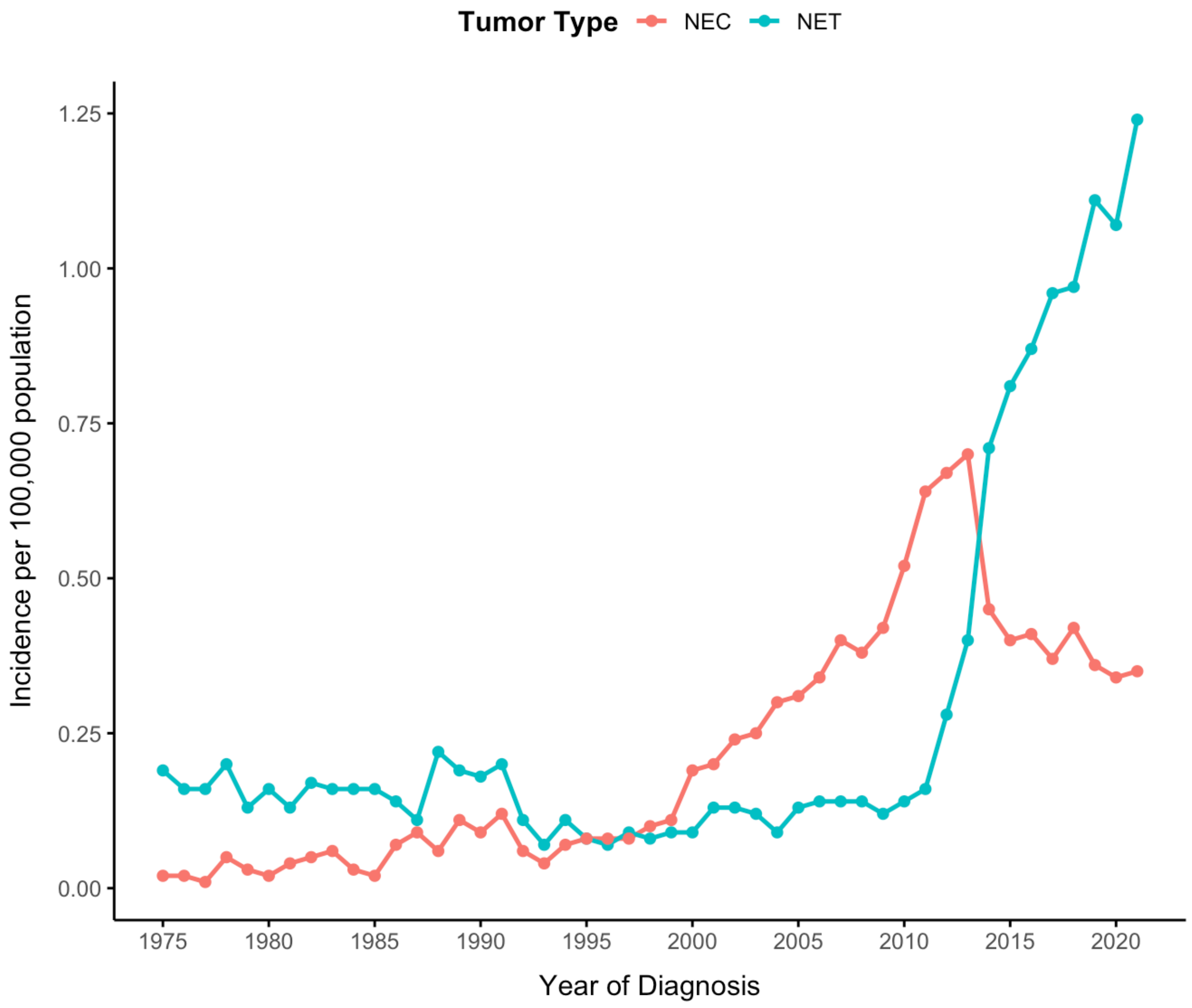

Figure S3. Median primary tumor size by year of diagnosis of pancreatic neuroendocrine neoplasms (NEN).

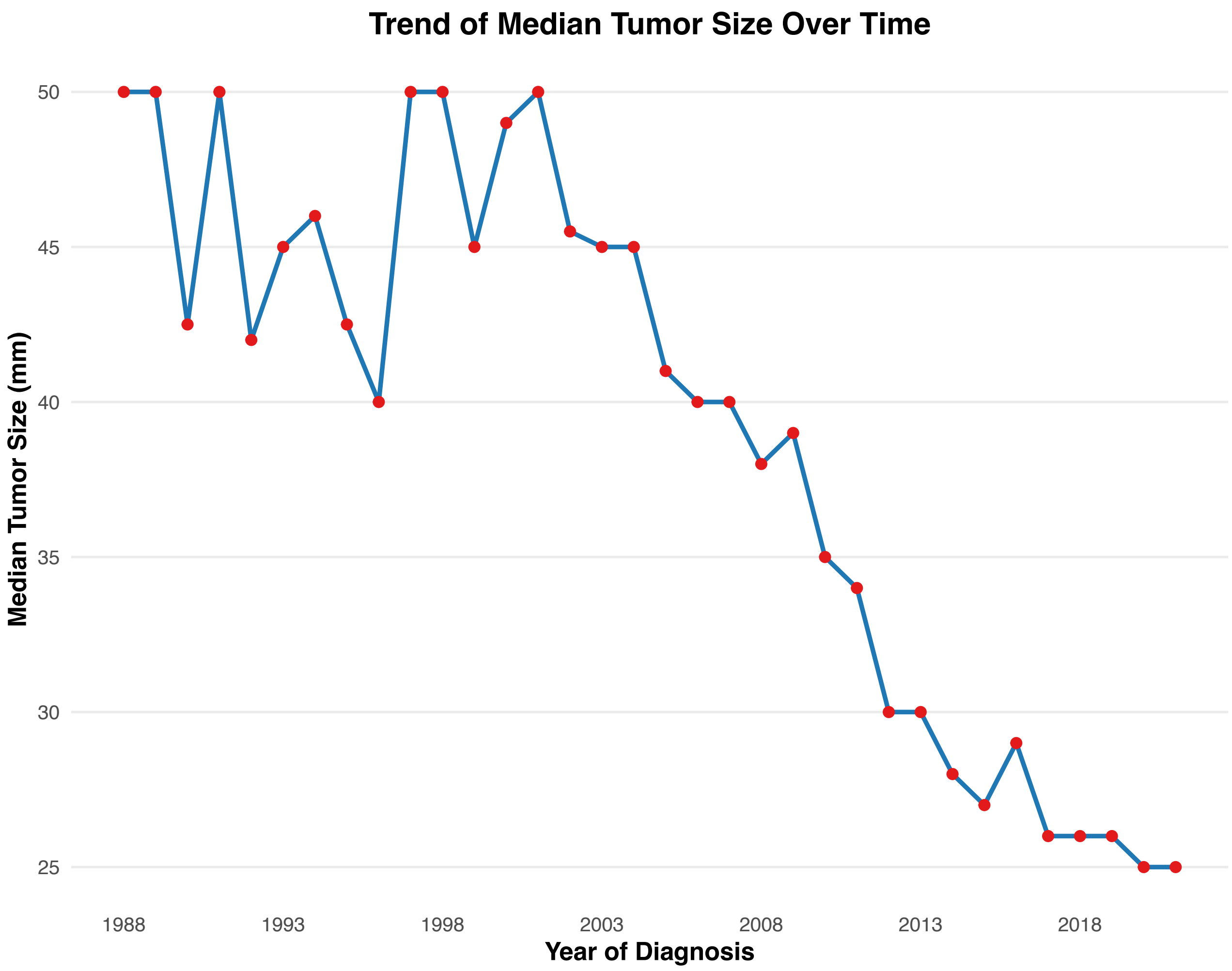

Supplement: Supplementary file 1 — Data S1. Supporting Information. [file JNE-38-e70136-s001.pdf]
